# Manipulating Connexin Communication Channels: Use of Peptidomimetics and the Translational Outputs

**DOI:** 10.1007/s00232-012-9488-5

**Published:** 2012-08-11

**Authors:** W. Howard Evans, Geert Bultynck, Luc Leybaert

**Affiliations:** 1 Institute of Infection and Immunity, Cardiff University School of Medicine, Heath Park, Cardiff, Wales CF14 4XN UK; 2 Department of Cellular and Molecular Medicine, K. U. Leuven Campus Gasthuisberg, 3000 Leuven, Belgium; 3 Department of Basic Medical Sciences, Physiology Group, Faculty of Medicine and Health Science, Ghent University, Ghent, Belgium

**Keywords:** Connexin hemichannels, Peptidomimetics, Clinical translation

## Abstract

Gap junctions are key components underpinning multicellularity. They provide cell to cell channel pathways that enable direct intercellular communication and cellular coordination in tissues and organs. The channels are constructed of a family of connexin (Cx) membrane proteins. They oligomerize inside the cell, generating hemichannels (connexons) composed of six subunits arranged around a central channel. After transfer to the plasma membrane, arrays of Cx hemichannels (CxHcs) interact and couple with partners in neighboring attached cells to generate gap junctions. Cx channels have been studied using a range of technical approaches. Short peptides corresponding to sequences in the extra- and intracellular regions of Cxs were used first to generate epitope-specific antibodies that helped studies on the organization and functions of gap junctions. Subsequently, the peptides themselves, especially Gap26 and -27, mimetic peptides derived from each of the two extracellular loops of connexin43 (Cx43), a widely distributed Cx, have been extensively applied to block Cx channels and probe the biology of cell communication. The development of a further series of short peptides mimicking sequences in the intracellular loop, especially the extremity of the intracellular carboxyl tail of Cx43, followed. The primary inhibitory action of the peptidomimetics occurs at CxHcs located at unapposed regions of the cell’s plasma membrane, followed by inhibition of cell coupling occurring across gap junctions. CxHcs respond to a range of environmental conditions by increasing their open probability. Peptidomimetics provide a way to block the actions of CxHcs with some selectivity. Furthermore, they are increasingly applied to address the pathological consequences of a range of environmental stresses that are thought to influence Cx channel operation. Cx peptidomimetics show promise as candidates in developing new therapeutic approaches for containing and reversing damage inflicted on CxHcs, especially in hypoxia and ischemia in the heart and in brain functions.

## Introduction

Gap junctions are cell–cell connections that ensure harmonious integration, regulation and equalization of metabolic events and signaling in tissues and organs. Their role in the coordination of cell behavior is vividly illustrated in the heart, where gap junctions in the intercalated discs provide pathways that allow direct intercellular electrical communication essential for synchronous contraction of component myocytes and for generating waves of rhythmic contractions observed in arteries. Gap junctions are constructed of paired connexin hemichannels (CxHcs), each composed of six protein subunits arranged around a central pore, and occur at adhesive areas where plasma membranes touch. Hcs are continuously recruited from surrounding unapposed plasma membrane areas and subsequently dock head to head with partners from adjacent cells and attach to the rims of preexisting gap junction plaques. The operational area and size of gap junction plaques where intercellular communication occurs are regulated by a balanced internalization and degradation of the dodecameric Cx channel units (Laird 2006; Goodenough and Paul 2009).

Many approaches have been applied to study the structure and function of gap junctions and their constituent CxHcs (Harris and Locke 2009). This account deals with the development of peptides that correspond to specific short Cx sequences that block widely the operation of gap junctions and CxHcs. The consequences ensuing from the actions of these peptides on Cx channels are allowing research to move forward into the realms of translational innovation across many fronts, especially in addressing the pathological consequences of ischemic stress that induces the channels to become leaky. Peptidomimetic approaches are complemented by gene knockout and antisense siRNA approaches to study Cx-based communication.

The development and application of short peptides mimicking sequences in various protein domains of Cxs, especially Cx43, initially focused on the two loops projecting outside the cell membrane; but peptides corresponding to sequences in the cytoplasmic intracellular loop and carboxyl tail are now finding application. Over the last 25 years, mimetic peptides have become important tools in elucidating a panoply of roles for gap junctions and their constituent CxHcs in a wide range of cells, tissues and organs. These are summarized in Tables 1, 2 and 3.

**Table 1:** Examples of the use of Gap26 and -27 mimetic peptides in studying the functions of gap junctions and connexin hemichannels in tissues/organs, cell layers and slices

Test model	Peptide	Effects	Reference
Arteries	Gap26/27	Block rhythmic contractions	Chaytor et al. (1997)
Mesenteric arteries	Gap27	Attenuates hyperpolarization	Dora et al. (1999)
Endothelium	Gap27	Attenuates Ach relaxations	Hutcheson et al. (1999)
Arteries	Gap26/27	Block EHF signaling	Chaytor et al. (2005)
Kidney	Gap27	Blocks renal vasodilatation	De Vriese et al. (2002)
Heart tissue	Gap26	Aids recovery after hypoxia	Hawat et al. (2010)
Heart lateral ventricle	Gap27^a^	Aids recovery after ischemia	Davidson et al. (2012)
Arteries	Gap27	Lowered intercell resistance	Matchkov et al. (2006)
Lung capillaries	Gap26/27	Inhibit Ca waves	Parthasarathi et al. (2006)
Trophoblasts/fibroblasts	Gap26/27	Block bilayer signaling and reduce DNA damage	Bhabra et al. (2009)
Various cell barriers	Gap27	Blocks signaling across barriers	Sood et al. (2011)
Brain endothelial and MDCK epithelial cells	Gap27	Inhibits Ca oscillations	De Bock et al. (2012)
Leukocytes	Gap27	Inhibits ATP release	Eltzschig et al. (2006)
Hippocampus	Gap27	Impairs learning, memory	Bissiere et al. (2011)
Hippocampus slices	Gap27	Inhibits epileptiform activity	Samoilova et al. (2008)
Rat amygdala	Gap27	Induces amnesia	Stehberg et al. (2012)
Spinal cord	Gap27^a^	Reduces swelling, reduces neuronal cell death	O’Carroll et al. (2008)
Optic nerve	Gap27	Attenuates CNS injury	Chew et al. (2010)
Hippocampus	Gap27^a^	Decreases cell death	Yoon et al. (2010)
Lung	Gap26	Reduces neutrophil transmigration	Sarieddine et al. (2009)
Various cells	Gap26	Blocks microtissue assembly	Bao et al. (2011)
Skin model systems	Gap27	Increased migration and proliferation	Pollok et al. (2011)

Gap26, VCYDKSFPISHVR; Gap27, SRPTEKTIFI

^a^Gap27 analogue. See Table 2 for sequence. A Gap27 acting on Cx40 channels (SRPTEKNVFIV) has been used on vascular tissues where this Cx is expressed

**Table 2:** Examples of use of Gap 26 and 27 mimetic peptides on various cells in culture

Test model/cells	Peptide	Effect	References
Skin fibroblasts, keratinocytes	Gap27	Increases migration in diabetes	Wright et al. (2012a, b)
HeLa Cx43 GFP	Gap26/27	Inhibit dye transfer	Berman et al. (2002)
Lymphocytes	Gap26/27	Inhibit transendothelial migration	Oviedo-Orta et al. (2002)
T/dendritic cells	Gap27	Cell sensitization abrogated	Ring et al. (2010)
Mesenteric smooth muscle	Gap27	Attenuates hyperpolerization	Dora et al. (1999)
Alveolar epithelial	Gap27	Inhibits Ca signaling	Boitano and Evans (2000)
Alveolar epithelial	Gap26/27	Inhibit dye transfer	Isakson et al. (2003)
Neonatal myocytes	Gap26	Inhibits ATP release in ischemia	Clarke et al. (2009)
HeLa/cardiac cells	Gap26/27	Inhibit Ca uptake and Ca waves	Verma et al. (2009b)
CD4^+^ T lymphocytes	Gap27	Inhibits T-cell proliferation	Oviedo-Orta et al. (2010)
B and T lymphocytes	Gap26/27	Decrease antibody production	Oviedo-Orta et al. (2010)
Corneal	Gap26	ATP release and Ca waves blocked	Gomes et al. (2005)
Ganglia	Gap27^a^	Limits retinal ganglion injury	Danesh-Meyer et al. (2012)
Neural retinal	Gap26	Limits ATP release and development	Pearson et al. (2005)
Astrocytes	Gap27	Abolished NMDA excitotoxicity	Froger et al. (2010)
Astrocytes	Gap26	Blocks glutamate release	Jiang et al. (2011)
Astroglia	Gap26/27	Block glutamate release	Orellana et al. (2011)
Glioma	Gap26/27	Delay apoptosis, cell death	Decrock et al. (2009a, b)
Astroglia	Gap26	Influences neural inflammation	Karpuk et al. (2011)
Glia	Gap26/27	Inhibit ATP release	De Vuyst et al. (2009)
Astroglia	Gap26	Inhibits ATP release and activation of P2Y receptors	Orellana et al. (2012a)
Astrocytes	Gap26/27	Induce anhedonia, depression	Sun et al. (2012)
Blood–brain barrier endothelium	Gap27	Inhibits ATP release and permeability of endothelium	De Bock et al. (2011)
AT11	Gap27	Inhibits Ca waves	Isakson et al. (2001)
Endothelium	Gap26	Inhibits ATP release	Robertson et al. (2010)
Bladder cancer	Gap26/27	Inhibit ATP release	De Vuyst et al. (2006)
T lymphocytes	1,848^b^	Blocks GJ docking	Mendoza-Naranjo et al. (2011)
Cardiomyocytes	Gap26	Blocks CxHc in cardiac hypoxia	Shintani-Ishida et al. (2007)
Platelets	Gap27	Blocks Cx 43/37 channels	Vaiyapuri et al. (2012)
Bone marrow stem cells	Gap27	Confirms Cx channels absent	Yang et al. (2009)

^a^VDCFLSRPTEKT peptide 5 derived from extracellular loop 2 of CxHc43

^b^Sequence of the Cx mimetic peptide not disclosed

**Table 3:** Effects of various intracellular Cx mimetic and other short and mainly Cx43 peptides on gap junctions and hemichannels

Test model	Peptide	Effect	Reference
Brain synapses	Carboxyl tail	Prevents Cx36/GJ formation	Flores et al. (2012)
Bladder cancer	Gap24^a^	Inhibits ATP release	De Vuyst et al. (2006)
Mouse hearts	Carboxyl tail^b^	Increases Cx43 and ps368 phosphorylation and induces arrhythmia	O’Quinn et al. (2011)
Heart	Carboxyl tail	May open gap junctions	Lewandowski et al. (2008)
Heart	R, any amino acid	May open gap junctions	Verma et al. (2009b)
Cardiac mitochondria	Gap27	Inhibits Cx43	Rottlaender et al. (2012)
T lymphocytes	Gap20^c^	Ineffective on gap junctions	Mendoza-Naranjo et al. (2011)
Endothelium- denuded arteries	Gap20^c^	Ineffective on gap junctions	Chaytor et al. (1997)
C6 glioma cells	L2 segment nonapeptide	Blocks CxHc but not gap junctions	Wang et al. (unpublished)
Corneal endothelial and C6 glioma cells	TAT-L2	Blocks CxHc but not gap junctions	Ponsaerts et al. (2010)
Basolateral amygdala	Cx43-L2 TAT	Blocks gliotransmitter release	Stehberg et al. (2012)
MDCK	CT9 peptide^b^ Carboxyl tail	Blocks Ca oscillations by removing high Ca closure	De Bock et al. (2012)

^a^Gap24: a Cx32 Gap20 homologue GHGDPLHLEEVK (from intracellular loop)

^b^Peptide RPRPDDLEI

^c^Gap20 EIKKFKYG

## Development and Exploitation of Cx Mimetic Peptides

Following the deduction of the complete amino acid sequences especially of Cx32 and Cx43, two of the 20 members of the Cx family of proteins, a number of short mimetic peptides were chemically synthesized and coupled to immunogenic carriers to generate antibodies to target specific domains and epitopes. The two highly conserved extracellular loops of Cx have proven to be poorly immunogenic, and it has been difficult to generate antibodies to these domains. Nevertheless, antibodies to both extracellular loop domains have been used in studies of a wide range of functions underwritten by Cx channels. These include (1) the topographical arrangement of Cx proteins in the membrane (Zimmer et al. 1987), (2) the roles of gap junctions in the development of mouse embryos (Becker et al. 1995), (3) Ca^2+^ wave signaling across cell layers connected by gap junctions (Boitano et al. 1998), (4) subcellular assembly of gap junctions (Rahman et al. 1993), (5) coordination of Ca^2+^ transients in beating cardiac myocytes (Verma et al. 2009b), (6) Cx43 as a candidate component of the immunological synapse (Mendoza-Naranjo et al. 2011), (7) the functional importance of the two exposed extracellular loops (Goodenough et al. 1988), (8) CxHc organization in polarized cells (Clair et al. 2008) and (9) tracking conformational changes as Cx traffic from the Golgi apparatus to gap junctions (Sosinsky et al. 2007). Antibodies to peptides from intracellular regions have been used extensively as diagnostic immunological/analytical tools and are widely available from commercial sources.

**Fig. 1:**
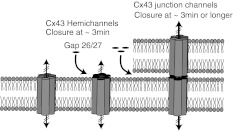
Mechanism of action of Gap26 and -27 mimetic peptides. Peptides bind to extracellular loop regions one and two, respectively, of CxHc, causing closure of channels within minutes. At later time intervals (30 min or longer) and depending on factors such as cell confluency, tissue, organ, tissue slice origin and thickness and conditions of perfusion of various organs, peptides permeate into intercellular spaces in gap junctions, causing disruption and diminished cell coupling. CxHcs with attached peptide move laterally toward the rims of gap junction plaques as they assemble and are then internalized

Although reagents such as heptanol, octanol, oleamide, lithium ions, quinine derivatives, carbenoxolone, fenamates, anandamide, oleamide, triarylmethanes and glycyrrhetinic acid inhibited gap junctional communication (Herve and Dhein 2010; Juszczak and Swiergiel 2009; Bodendiek and Raman 2010), there remained a need for more specific reagents with a known mechanism of action. From early on, it became evident that Cx mimetic peptides used to generate the antibodies to gap junctions might prove useful as chemical tools to manipulate channel operation; it was argued that the utility of antibodies was restricted by their size and limited penetration across the cell membrane and into intercellular regions where gap junctions are located. Such drawbacks would be overcome by using small mimetic peptides that could penetrate into intercellular junctions, disrupt the docking and/or operation of hemichannels and, thus, target the gap junction (Fig. 1).

Two studies using model systems marked the beginning of the exploration of Cx mimetic peptides as tools to study gap junction functions. The first took advantage of the contractile behavior displayed by embryonic chick heart myoballs and known to require coordination provided by intercellular communication via gap junctions. The effects of a series of 15 Cx peptides, corresponding to short sequences mainly in intra- and extramembrane amino acid regions of the tetraspan membrane protein in delaying gap junction functions, were determined (Warner et al. 1995). A parallel study that used six dodecapeptide Cx mimetics to interrupt communication across gap junctions generated in *Xenopus* oocytes transfected with RNA to Cx32 (Dahl et al. 1994) likewise pointed to the potential of using short peptides to tamper with Cx-dependent intercellular communication. Warner et al. (1995) pinpointed motifs that included short sequence motifs, SRPTEK in extracellular loop 1 and SHVR in extracellular loop 2, as likely potent peptides for use in disrupting cell communication. These motifs were later incorporated into Gap26 and -27 mimetic peptides and their close homologues (see Tables 1, 2, 3). Kwak and Jongsma (1999) used dye coupling and dual patch-clamp approaches to study the inhibition of Cx channels using peptide mimetics from the second extracellular loop of Cx43 and Cx40. An extensive literature has since built up around the study of Cx-dependent cell communication processes, especially with peptides mimicking sequences in Cx43 and, to a lesser extent, Cx40 and Cx37, which are widely expressed in the vascular system as well as with Cx32 (De Bock et al. 2011). Gap26 and -27 have emerged as mimetic peptide tools that have entered the literature in studies that explore the operation and function of Cx channels in several settings (Tables 1, 2). As discussed below, the blockage of direct cell coupling across gap junctions (Evans and Boitano 2001) is now likely to be a secondary event that follows initial interaction of the peptides with CxHcs. Recent work is increasingly focused on the translational and therapeutic possibilities offered by the action of the mimetic peptides, especially in averting or reversing tissue damage in ischemia and inflammation.

## Gap Junctions and CxHcs

The view that CxHcs possess functions in their own right and are able to operate in different modes from gap junctions has now become generally accepted (Goodenough and Paul 2003; Bennett et al. 2003; Evans et al. 2006). CxHcs were detected in *Xenopus* oocytes (Ebihara and Steiner 1993), a test bed to study gap junction expression and function and where the channels were observed to open in low-Ca media. Hc opening was also detected in vertebrate retinal dendrites (Malchow et al. 1993). These early studies appeared against the background view that CxHcs sustained in open configuration in membranes would lead to potentially catastrophic cellular outcomes by allowing transmembrane escape from cells of small intracellular signaling molecules, e.g., ATP and glutamate, and would result in a collapse or dissipation of ionic gradients. The possible importance of CxHcs operating under normal physiological situations in cells and tissues was critically evaluated (Spray et al. 2006). Collateral evidence for the functional reality of CxHcs began to appear later for roles in pathology with, e.g., the demonstration that leaky mutated CxHcs in the ear were linked to deafness (Stong et al. 2006; Scott and Kelsell 2011) and a mutation in the intracellular loop of Cx43 that decreased single-channel conductance and is linked to neurological disturbances in oculodentodigital dysplasia (Lai et al. 2006). Reconstituted Hcs were used to investigate the influence of Ca and atomic force microscopy, to study Hc pore opening (Thimm et al. 2005). It is now generally accepted that CxHcs open under environmental circumstances considered to be stressful to cells such as volume or osmotic changes; oxidative, metabolic and mechanical stresses; and especially hypoxia/ischemia.

**Fig. 2:**
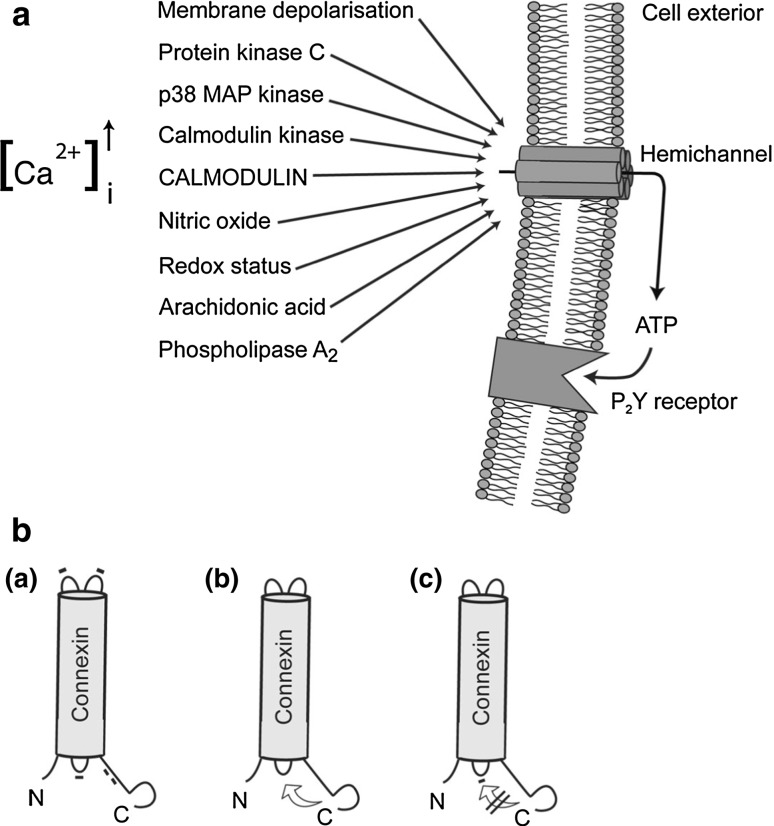
**a** Several intracellular signals and events influence CxHc functions. Membrane depolarization above +30 V opens Hc. Several kinases may also be involved; PKC closes Hc, while p38MAPK and calmodulin kinases result in Hc opening. The cytoplasmic Ca concentration ([Ca^2+^]_i_) is also an important modulator; below 500 nM [Ca^+^] calmodulin is a key Ca binding protein and specific binding sites are present on Cx. An increase in intracellular Ca causes Hc opening, while Hc activation is lost at higher concentrations. Arachidonic acid stimulates Hc opening, and amino acid metabolites generated by PLA_2_ activation may contribute to this. Nitric oxide and oxidative stress also result in opening of Hc. Figure modified from De Vuyst et al. (2009). **b** *a* Sites on the exposed extramembrane regions of Cx43 where the mimetic peptide sequences originated. *b* Proposed intramolecular mechanism of CxHc gating involving interaction of the carboxyl tail with the intracellular loop. *c* Binding of a nonapeptide mimetic derived from L2, the Cx43 intracellular loop region to a site on the carboxyl terminus regulates the closure of CxHc and leads to blockage of the channel

If not contained, leaky CxHcs can lead to apoptosis and cell death (Saez et al. 2010; Decrock et al. 2009a, b). Binding of mimetic peptides such as extracellular loop peptides Gap26 and -27 to the extracellular face inhibited functions associated with CxHcs; these are also regulated by membrane depolarization, phosphorylation of several sites on the carboxyl tail of Cx43 (Solan and Lampe 2009), S-nitrosylation (Retamal et al. 2006) and SUMOylation of lysine residues in the intracellular loop (Kjenseth et al. 2012). The particle–receptor hypothesis (see below) explained the mechanics of gating in those Cxs with extended cytoplasmic tails such as Cx43, Cx40 and Cx45 (Delmar et al. 2004). Recent evidence suggests that mimetic peptide perturbation of intracellular domains, especially the interactive cytoplasmic loop (CL) and the carboxyl tail (CT), also influences Hc functions (Ponsaerts et al. 2010). CxHc gating is also conditioned by signaling cascades operating in subplasmalemmal environs (Fig. 2).

Besides structural differences, with Hcs being asymmetrical and gap junctions being symmetrical double channels, there are other important differences between the functions of these weakly selective channels. CxHcs stand out in being responsive to environmental changes and contingencies. Gap junction channels allow the cytoplasms of cells to be linked directly, whereas open CxHcs provide channels connecting the cell’s external environment with the cytoplasmic mileu beneath the plasma membrane. In a cell-signaling context, changes in CxHc open probability provide a mechanism for paracrine intercellular communication by allowing small molecules such as ATP and K to exit and Ca to enter cells. ATP release underwrites purinergic intercellular signaling (Isakson et al. 2001; Paemeleire et al. 2000; Kang et al. 2008), a function shared with pannexin channels.

Gap junction channels exist normally in open mode, and an elevation in intracellular Ca^2+^ leads to closure (generally 500–2,000 nM). In contrast, CxHcs open at 500 nM (De Vuyst et al. 2006, 2009, 2011). Differences in Ca sensitivity of the two channel types may relate to their complementary roles in regulating intracellular Ca oscillations and the intercellular propagation of Ca waves (Verma et al. 2009a; Orellana et al. 2012b; De Bock et al. 2012). The various intracellular processes that influence the gating of Cx43Hcs are shown in Fig. 2b. Gap junctions and CxHcs respond differently to lipopolysaccharide and basic fibroblast growth factor, a consequence of their involvement in releasing ATP (De Vuyst et al. 2007); their channel functions also respond differently to many growth factors (Schalper et al. 2012).

## Widespread Use of Gap26 and Gap27 Mimetic Peptides

Cx43 is by far the most widely distributed Cx in tissues and organs. It is therefore not surprising that Gap26 and -27, derived from Cx43 sequences, have found extensive use. A modified Gap27 peptide (Table 1) incorporating a sequence mimicking that in Cx40 has also proven useful in vascular tissues and other tissues where both Cxs are present (Chaytor et al. 1999; Wright et al. 2009). Early studies showed that inhibition by mimetic peptides was largely reversible as assessed by intercellular transfer of small fluorescent “reporter” dyes of varying size and by measuring the extent of Ca wave propagation over multiple rows of cells (Boitano and Evans 2000).

As the operational presence of CxHcs became evident, inhibition by mimetic peptides of ATP release in a wide range of tissues and cells (Tables 1, 2), of glutamate in astrocytes (Yang et al. 2009) and astrocytes/microglia (Orellana et al. 2011) and of ion trafficking, especially Ca^2+^ entry, were observed. Uptake of reporter dyes across CxHcs became a reliable and routine method to demonstrate open or leaky CxHcs (Li et al. 1996). A range of other effects were also noted on prolonged exposure (20 min or longer) to the mimetic peptides such as smooth muscle contraction in endothelium-denuded arteries (Chaytor et al. 1997; Hutcheson et al. 1999) and immunoglobulin production by lymphocytes (Oviedo-Orta et al. 2001). Subsequently, as the presence of CxHcs widened, it became increasingly clear that the primary action of Gap26 and -27 is in fact directed at CxHcs. Nevertheless, a combination of direct signaling across gap junctions and paracrine signaling acting through metabotropic purinergic receptors following release of ATP (Fig. 2) provides scope for complementary intercellular interplay involving both routes in several settings (Anselmi et al. 2008; Cotrina et al. 1998).

Initial experiments showing inhibition by mimetic peptides of CxHcs were carried out mainly using cultured cells (Table 2), followed by numerous examples in various tissues and organs, cell layers and thin tissue slices (Table 1). The growing attribution of nonjunctional properties to CxHcs (Cotrina et al. 2008; Kameritsch et al. 2011) has led to studies that attempt to unravel the potential functions in the realms of adhesion and cell movement. Enhancement of migration of dermal fibroblasts after treatment with Gap27 pointed to nonjunctional roles for CxHcs in cell movement and extending, e.g., to assessing their potential translational application in addressing their efficacy in accelerating wound healing in diabetes and in a range of micro-/macrovascular diseases (Wright et al. 2009, 2012a; Pollok et al. 2011). Gap27 increased migration of human keratinocytes and dermal fibroblasts, and the efficacy of the mimetic peptide was different in euglycemia and hyperglycemia (Wright et al. 2012b). Gene array approaches indicated that Gap27 induced upregulation of genes involved in extracellular matrix remodeling and cell adhesion. Exposure of cells to Gap27 may have effects on Cx43 phosphorylation, especially of serine 368; phosphorylation of this amino acid decreases Cx43 channel activity (Solan and Lampe 2009) and is a key process in the gating of Cx43 channels. Many of the effects of the mimetic peptides were seen after long-term exposures. Cx43 has been detected in the inner mitochondrial membrane of heart cells and has been implicated in cardiac preconditioning (Boengler et al. 2009; Rottlaender et al. 2012). The opening of mitochondrial CxHc influences K^+^ fluxes in processes that are linked to cardioprotection. The functions of Cx43Hc at this location are open to study using mimetic peptides developed to Cx43 cytoplasmic sequences (Verma et al. 2009a).

## Mechanism of Action of Cx Mimetics

To further develop and refine the actions of Cx mimetics, it is important to gain insight into the mechanisms by which they block Cx channels. A major advance was the demonstration that the primary action of Gap26 and -27 was likely to be on CxHcs prior to blockage of gap junctions. With the acceptance that CxHcs and gap junctions were targets but in different time frames, the effects of the peptides on both were examined using electrophysiological approaches. These had already proven useful in studying electrical coupling across gap junctions in cell pairs and the electrical properties of CxHcs (Harris 2001; Contreras et al. 2003; Saez et al. 2005). Using a voltage-clamp approach in nonconfluent and confluent HeLa cells expressing Cx43, Gap26 inhibited macroscopic currents through CxHcs in 2–3 min. In contrast, electrical coupling in cell pairs was delayed and completely inhibited after 30 min or more (Desplantez et al. 2012), indicating direct action of Gap26 on an exposed extracellular loop, as previously suggested by studies of CxHcs inserted into lipid bilayers (Liu et al. 2006). The extended time course of action on gap junctional electrical coupling suggests that longer diffusion pathways into plasma membrane junctional domains lead ultimately to blockage of gap junctional coupling (Fig. 1). The changes in conductance and voltage gating of CxHcs by Gap26 suggested interaction of the peptide at a consensus sequence in the first extracellular loop that may contribute to the inner wall of the pore (Sosinsky and Nicholson 2005), resulting in a decrease in channel diameter in the extracellular vestibule. Desplantez et al. (2012) also proposed that, of the two voltage gates controlling Hc, the slow gate is the more likely to be influenced by the binding of Gap26.

An expanded complementary study by Wang et al. (unpublished) examined the mechanism of action of Gap26 and -27 in HeLa cells stably transfected with Cx43 and in pig ventricular myocytes endogenously expressing Cx43 using a voltage-clamp approach. Both mimetic peptides inhibited Cx43Hc unitary currents within minutes. An important outcome of this study with cardiac translational implications was that unitary current activity was promoted by a moderate elevation of cytoplasmic Ca, an event observed in cardiac arrhythmogenesis.

Gap26 and -27 have been used mainly to inhibit Cx43 channels, but actions on other Cx channels have also been studied. For example, Wright et al. (2009) carried out an extensive study of the effects of Gap26 and -27 on dye coupling in cells expressing several human Cxs and found that Gap27 had broader Cx specificity than Gap26. In some situations where multiple Cxs are expressed, as in skin, broad specificity can be an advantage.

The precise protein domains to which Gap26 and -27 mimetic peptides attach and any conformational changes induced in Cx channels remain to be determined. Fluorescently labeled Gap26 and -27 are poorly soluble; but evidence points to attachment and retention at cell exteriors, and problems of image resolution could not answer the issue of whether attachment of a mimetic peptide ligand to a “channel receptor” sequence can lead to peptide internalization (Evans, unpublished work). Voltage-clamp approaches have been useful in deciphering the inhibitory action of these peptides on Cx channels and go a long way toward resolving questions concerning the specificity of mimetic peptide inhibitory effects. Cx mimetics have been claimed as effective inhibitors due to steric pore block (Wang et al. 2007). Steric block may indeed occur, but recent data indicate that it only occurs when peptides are used in the range of 1 mM and above (Wang et al. unpublished). The examples shown in Tables 1 and 2 used mainly protein concentrations of 100–250 μM. In the vast majority of these studies, investigators have used scrambled peptides or short peptides derived from regions believed not to be directly involved in channel operation (often internal sequences) as controls and, in each instance, they pointed to a high sequence dependence of mimetic peptide action in blocking gap junctional coupling and, more recently, ATP release or dye entry across CxHcs.

Pannexins, of which three are identified compared to around 22 in the Cx family of proteins, also form oligomeric channels with a similar tetraspan topographical arrangement in the membrane to CxHcs. However, these two protein families share no amino acid homology. Pannexins, unlike Cxs, are posttranslationally glycosylated (D’Hondt et al. 2009; Scemes et al. 2009) and are disinclined to dock with partner pannexins on neighboring cells. It follows that they are not expected to generate gap junction-like structures and, indeed, are found at higher levels inside cells (D’Hondt et al. 2011). In contrast, a recent report claims that they can form gap junctions and act as Ca leak channels in the endoplasmic reticulum (Ishikawa et al. 2011). Pannexins are relatively unaffected by changes in cytoplasmic Ca^2+^ levels and, unlike Cx, are calmodulin-insensitive. Pannexins are especially abundant in neural tissues. The action of Gap26 and -27 on pannexin channel currents has not yet been rigorously tested. It is worth noting that high sequence diversity occurs in the intracellular loop of Cx proteins; the carboxyl tail of larger Cx proteins also varies in length, sequence and the extent of posttranslational modification.

Small peptides generally have little structural organization but may assume some on binding to a target that then becomes subject to conformational change. Studies of the interaction of calmodulin with mimetic peptides from the intracellular loop of Cx43 demonstrate a way forward toward functionally dissecting this key region of Cxs (Myllykoski et al. 2009). A calmodulin binding region in Cx43 was located to amino acid residues 136–158 (Zhou et al. 2007). Clearly, further knowledge of the structural organization of Cx43Hc is awaited along the lines available on CxHc26 (Maeda et al. 2009; Oshima et al. 2011).

Table 3 lists the growing number of mimetic peptides derived from the CL and CT that act on CxHcs and gap junctions. Many of these peptides are not able to cross the plasma membrane. To gain access to their sites of action, many of these mimetics need to be attached first to “Trojan” cell-penetrating peptides that contain a membrane-translocation motif (Jarver and Langel 2006). Intracellular loop mimetic peptides with a positive charge due to several lysine residues stand out as candidates, for they can, depending on length, gain access to intracellular targets without need for attachment of a membrane-penetrating peptide or by microinjection, as discussed below. Indeed, peptides of around 1 kDa or less may be able to access the cytoplasm via open CxHcs.

## Mimetics and Other Cx Peptides as Agents to Address Pathology

In a cardiac context, blockage by Gap26 and -27 of gap junctional coupling between myocytes negated their candidacy for use, especially in therapeutic approaches to address cardiac arrhythmia, for the outcomes of peptide treatment were likely to compound the pathology. Consequently, studies in heart focused on nonmimetic hexapeptides shown to be prorhythmic, especially ZP123 derived from AAp10 and later renamed rotigaptide (Hagen et al. 2009; Herve and Dhein 2010). This hexapeptide promoted changes to Cx phosphorylation, increased gap junctional coupling (Clarke et al. 2006) and showed promise in animal models, where it prevented ventricular tachycardia in myocardial ischemia (Xing et al. 2003). However, following inconclusive clinical trials, rotigaptide was not developed further. Cellular studies showed that despite its prorhythmic action on gap junctions, ZP123 also led to the opening of CxHcs, causing ATP release. In a cardiac myocyte ischemia model, Gap26 blocked the release of ATP, whereas ZP123 enhanced its release across Cx43Hc (Clarke et al. 2009). Other antiarrhythmic peptides—such as Gap134, a prorhythmic dipeptide—were also developed (Hennan et al. 2009).

In the meantime, encouraging outcomes in addressing ischemic cardiomyopathy using Gap26 have appeared. These results can be explained by the direct action of this mimetic peptide on CxHc rendered open under hypoxic/ischemic conditions. Hawat et al. (2010) showed that following myocardial ischemic insult, Gap26 helped to confer protection through its binding to and blockage of Cx43Hc. Similar protection from hypoxic stress by these peptides has also been reported. Vascular leak and retinal ganglion cell death were reduced by application of a close homologue of Gap27 in an atrial ischemia model where Cx43Hc expression was increased (Danesh-Meyer et al. 2012). The same group also reported that infusion of Gap27 in a large animal model with cerebral injury delayed the onset of ischemic injury and suggested that Gap27 homologue peptides reduced inflammation. For example, mimetic peptide treatment reduced the spread of damage after traumatic spinal cord injury where Cx43Hc plays a critical role (O’Carroll et al. 2008; Huang et al. 2012). Such studies combine to show that Gap26 and -27 mimetic peptides acting on CxHc-related functions, especially in situations of tissue injury, show therapeutic potential. In brain, inhibition of inflammation-induced activation of Cx43Hc in astroglial cells was attenuated by treatment with Gap26 and -27, suggesting that the channels play a critical role in instigating neuronal death and pointing to a neuroprotective role for these mimetic peptides (Froger et al. 2010). Interaction of the mimetic peptides with CxHcs is also likely to modify intracellular signaling cascades in subplasma membrane environments (Fig. 2a).

The action of Gap26 and -27 on CxHcs and later at gap junctions encouraged the view that pathological outcomes could be fine-tuned if mimetic peptides were to become available that confined their blocking action on CxHcs resident in the plasma membrane. Attention had already focused on the intracellular loop region implicated in explaining gap junction gating in cardiac cells and involving intramolecular interaction of the carboxyl tail region with the intracellular loop L2 region (a region in Cx43 incorporating amino acids 119–144) and described as the particle–receptor hypothesis (Delmar et al. 2004). The role of the L2 domain as a key molecular determinant of Cx43 function originated from the acidification-related closure of gap junctions (Delmar et al. 2004), and the particle-receptor hypothesis has been proposed to explain Cx43 closure during acidification, with the CT moving as a flexible gating particle that binds to the L2 site at the intracellular vestibule and leading to closure of the gap junction pore. Because of the similarity to the inactivation of K^+^ channels, this mechanism has also been called the ball and chain model of gap junction closure. A 34-amino acid nonmimetic peptide (RXP-E) and its derivatives were developed to target the Cx43CT (Shibayama et al. 2006; Lewandowski et al. 2008; Verma et al. 2009a). When coupled to cell-penetrating peptides, these composite peptides successfully restored gap junctional coupling and impulse propagation in cultured neonatal rat ventricular cardiomyocytes. Although the ability of L2 peptide to bind to the Cx43CT region has been intensively studied, less is known about how it is influenced by pH.

Loop–tail interactions also control Cx43Hc opening (Ponsaerts et al. 2010). The interaction of a cytoplasmic loop domain with the C-terminal region is an important requisite for the opening of Cx43Hc in response to stimuli such as lowering of extracellular Ca^2+^ or increasing intracellular Ca^2+^. In contrast, Cx43 gap junctions behave in the opposite manner and are closed by intramolecular loop–tail interactions (Delmar et al. 2004; Hirst-Jensen et al. 2007). Interfering with loop–tail interactions can inhibit the operation of Cx43Hc. One way to suppress CxHc operation in response to high intracellular Ca^2+^ is to activate the actomyosin contractile system, a process that appears to physically dislodge the CT from the CL region (Ponsaerts et al. 2012). Importantly, the selective myosin11-ATPase inhibitor blebbistatin restores Cx43Hc activity when cells are exposed to high intracellular Ca (Ponsaerts et al. 2012). The proteins involved in this mechanism linking Cx43Hc and the actomyosin contractile system have not been identified, but a likely target is the CT region of Cx43Hc, for Cx43 lacking the CT is inactive. Even in the absence of a functional actomyosin cytoskeleton, loop–tail interactions in Cx43Hc can be disrupted using mimetic peptides from the L2 region, and coupling the peptides to a TAT cell-penetrating sequence inhibits CxHc opening. An important interactive target of L2-region peptides is the last 10 amino acids of the carboxyl tail (CT10). Direct binding between the L2 domain and CT10 has been demonstrated by surface plasmon resonance. Thus, TAT-L2 allows exploitation of the opposite regulation of gap junctions and Cx43Hc and can provide a route to selectively inhibit Cx43Hc while maintaining gap junctional communication. The importance of the 10–amino acid terminal carboxyl domain has been studied in detail also using electrophysiological approaches in *Xenopus* oocytes where the TAT-L2 and TAT-CT10 peptide constructs have been crucial in analyzing and deciphering intramolecular loop–tail interactions (Ponsaerts et al. 2010, 2012). These studies illustrate how mimetic peptide approaches for selectively studying the physiological functions of Cx43Hc can complement knockdown/knockout approaches in, e.g., processes leading to cell death (Decrock et al. 2011) and in brain functions in the basolateral amygdala, where Cx mimetic peptides such as Gap27 demonstrate that Cx43Hc activities are implicated in amnesia (Stehberg et al. 2012).

A short nonmimetic peptide (designated RXP-E) that bound to the carboxyl tail of Cx43, derived from heart lysates and studied in animal and cellular models, prevented action potential block in the heart (Lewandowski et al. 2008). A series of nonmimetic peptides with a motif designated RXP and containing a predominance of basic amino acid increased the mean open time of gap junction channels; these peptides were proposed as potential functional regulators in ischemia-induced arrhythmias (Shibayama et al. 2006; Verma et al. 2009a).

Currently, the application of novel short peptides mimicking sequences in the L2 region of Cx43 (Delmar et al. 2004) where a putative calmodulin binding site is identified (Zhou et al. 2007) proceeds. One major aim is to design mimetic peptides that confine their blocking actions to CxHcs with no interference on gap junction functionality, thus avoiding pro-arrhythmic consequences and allowing the bound mimetic peptide to arrest, e.g., the loss of vital cell metabolites via CxHcs in cardiomyocytes subject to hypoxia or ischemia perfusion injury. A potential tool for more selective inhibition of Hcs without associated inhibition of gap junctions is a synthetic mimetic peptide corresponding to the L2 region that, when delivered into the cell by a whole-cell recording pipette, prevented gap junctional opening to the subconductance state at high transjunctional voltage and increased the channel open time (Ponsaerts et al. 2010). Also, L2 peptide linked to the TAT membrane translocation motif (TAT-L2) inhibited Cx43Hc activation, further suggesting that prevention of interaction of CT and CT suppresses Hc opening. Consequently, the particle–receptor hypothesis described above has an opposite outcome for Hcs compared to gap junctions. This raises questions concerning why this is so, for the composite proteins of the channels are identical but the Ca sensitivity of the Cx43Hc and gap junctions differs. It now appears that intracellular interactive partners may also be different at the cytoplasmic aspects of junctional and nonjunctional regions of the plasma membrane. One possible reason for these differences is that Hcs interact with the actomyosin contractile system, causing dynamic loop–tail interactions that control Hcs that differ from those occurring at gap junctions (Ponsaerts et al. 2012).

New mimetic peptides derived from the carboxyl tail of Cx43 are also being assessed. Here, peptide mimetic design has to deal with a region where the CT interacts with cytoskeletal elements (Herve et al. 2011; Palatinus et al. 2011). This region also incorporates several phosphorylation sites that condition channel gating (Solan and Lampe 2009). In the heart, the cytoskeletal adaptor protein ankyrin-G interacts with Cx43 and is a likely key intercalated disc complex in the pathophysiology of arrhythmias (Sato et al. 2011). A short Cx43-CT peptide incorporating the last nine amino acids and linked to a cell permeabilization sequence inhibited pathological changes at gap junctions that are related to ventricular arrhythmias (O’Quinn et al. 2011). This peptide disrupted the interaction between the PDZ domain of ZO1 and Cx43, thus accelerating assembly of gap junctions from a precursor pool of Hcs (Hunter et al. 2005; Rhett and Gourdie 2012). The peptide also enhanced PKC-epsilon-associated phosphorylation at the Cx43-S368 site, an effect that is activated in ischemic preconditioning and can reduce cardiac injury (Ek-Vitorin and Burt 2012; Srisakuldee et al. 2009). A similar peptide with the same sequence named CT9 prevented high Ca–induced closure of Cx43Hc (De Bock et al. 2012; Ponsaerts et al. 2012). In the same vein, a 15–amino acid mimetic peptide derived from a sequence in the CT of Cx36 modified gap junction conductance in goldfish electrical synapses, and this peptide was injected intradendritically (Flores et al. 2012).

## Concluding Comments

Peptides that mimic short sequences in extramembrane domains of Cx proteins show huge potential in addressing Cx-based communicationopathies by pharmacological means. The functions attributed to Cx, especially Cx43, continue to expand and include not only roles in intercellular cell communication effected by CxHc and gap junctions but also cell adhesion, cell migration and mitochondrial inner membrane channel functions. It is likely that the range of Cx mimetics and their applications will increase, for they provide activation mechanisms for manipulation of intercellular signaling and communication. Furthermore, the peptides can also help cells protect themselves from events emanating from leaky channels, events that may ultimately lead to apoptosis. Intriguingly, in the brain the Gap26 and -27 mimetic peptides that act mainly on astrocyte Cx channels have been shown to affect depression (anhedonia), epileptiform activity, memory consolidation and amnesia, further emphasizing the importance of Cx-mediated communication and signaling in a wide range of settings.
